# Collagen Self-Assembly on Orthopedic Magnesium Biomaterials Surface and Subsequent Bone Cell Attachment

**DOI:** 10.1371/journal.pone.0110420

**Published:** 2014-10-10

**Authors:** Nan Zhao, Donghui Zhu

**Affiliations:** 1 Department of Chemical, Biological and Bio-Engineering, North Carolina Agricultural and Technical State University, Greensboro, North Carolina, United States of America; 2 NSF Engineering Research Center-Revolutionizing Metallic Biomaterials, North Carolina Agricultural and Technical State University, Greensboro, North Carolina, United States of America; University of Akron, United States of America

## Abstract

Magnesium (Mg) biomaterials are a new generation of biodegradable materials and have promising potential for orthopedic applications. After implantation in bone tissues, these materials will directly interact with extracellular matrix (ECM) biomolecules and bone cells. Type I collagen, the major component of bone ECM, forms the architecture scaffold that provides physical support for bone cell attachment. However, it is still unknown how Mg substrate affects collagen assembly on top of it as well as subsequent cell attachment and growth. Here, we studied the effects of collagen monomer concentration, pH, assembly time, and surface roughness of two Mg materials (pure Mg and AZ31) on collagen fibril formation. Results showed that formation of fibrils would not initiate until the monomer concentration reached a certain level depending on the type of Mg material. The thickness of collagen fibril increased with the increase of assembly time. The structures of collagen fibrils formed on semi-rough surfaces of Mg materials have a high similarity to that of native bone collagen. Next, cell attachment and growth after collagen assembly were examined. Materials with rough surface showed higher collagen adsorption but compromised bone cell attachment. Interestingly, surface roughness and collagen structure did not affect cell growth on AZ31 for up to a week. Findings from this work provide some insightful information on Mg-tissue interaction at the interface and guidance for future surface modifications of Mg biomaterials.

## Introduction

There is an increasing interest in magnesium (Mg)-based alloys as implantable orthopedic medical devices because of their biodegradability and good biocompatibility [1]–[11]. Compared with other metal biomaterials, e.g., stainless steel, titanium alloys, and cobalt-chromium alloys, Mg alloys have several advantages for orthopedic application. First, their physical and mechanical properties including density (1.74–2.0 g/cm^3^), elastic modulus (41–45 GPa), and compressive yield strength (65–100 MPa), are much closer to that of natural bone, and therefore can avoid the stress shielding effect [12]–[14]. Second, Mg is an essential element for many biological activities including enzymatic reaction, formation of apatite, and bone cells adsorption [15]. Third, Mg alloys can eliminate the necessity of a second surgery to remove the permanent bone implants.

The success of an medical implant is largely dependent on the interaction between the surface of the implant and the surrounding tissues [16]. Both surface chemistry and topography of implants can affect biological activities such as osteoblasts metabolism, collagen synthesis, and alkaline phosphatase activity [17]–[20]. Cells often display distinctive morphological and metabolic properties when they are in contact with materials with different surface roughness [19]. It is a general consensus that cells cannot directly recognize bare metal materials in vitro or in vivo. It is the biomacromolecules absorbed on metal materials serve as a bridge connecting cells to the solid surface [20]. Therefore, the adsorption of ECM proteins and subsequent structure changes may lead to different cell fate. Collagen as the most abundant ECM protein is the major component of natural bone. It plays an important role in cell attachment, mechanical support, and apatite nucleation [21]. The mean weight percent of collagen in modern mammal bone is around 20.8%, and 90% of the organic matrix in bone is comprised of collagen [22], [23]. Studies have been carried out in the past with respect to the self-assembly characteristic of collagen [24]–[26] and application of type I collagen as coating materials [27]–[29]. Fang et al. showed that different mica surfaces affect D-period during collagen self-assembly [30]. Nassif et al. reported that collagen-apatite matrix is necessary for organization of collagen fibrils into 3-D scaffolds and nucleation of hydroxyapatite [31]. However, the information on collagen and Mg biomaterial interaction is still missing in the literature.

Previous studies showed that biodegradable Mg alloys enhanced bone-implant strength and osseointegration compared to titanium alloys [2], [32]. With the increasing orthopedic applications of Mg alloys, there is an urgent need to fill such a gap to understand how collagen molecules interact with the solid metal phase at the interface as well as the subsequent cell attachment. Evaluating the interaction between collagen and Mg implant in vivo could be very challenging currently owing to the complexity of biological system. Hence, an in vitro model was developed here to study type I collagen adsorption, assembly and osteoblasts adhesion on different Mg materials.

## Materials and Methods

### Material Preparation

High Purity Mg extrusion rod and MgAlZn (AZ31) extrusion rod with diameter of 10 mm were obtained from Goodfellow, USA. Materials were cut (Techcut 5, Allied High Tech Products, USA) into D10×1 mm disc and polished with SiC paper up to 1200 grit. All materials were supersonically cleaned (M2510 Ultrasonic cleaner, Branson, USA) in acetone (Fisher Scientific, USA) and washed 3 times with ethanol (Fisher Scientific, USA) followed by 30 minutes UV sterilization (1380 Biological Safety Cabinet, Thermo, USA). For each experiment, at least 3 replicates were used.

### Collagen assembly on Mg surface

#### The effect of collagen concentration

Rat tail type I collagen solution (Life Technologies, USA) of 3 mg/ml was diluted by D-phosphate-buffered solution (DPBS, Invitrogen, USA) to 10, 50, 100, and 200 µg/ml, respectively. 50 µl final collagen solutions (pH of 7) were spread on testing materials surface followed by incubating under 37°C for 2 hours and then dehydrate with ethanol gradient. The morphology of final collagen structure was characterized by Scanning Electron Microscope (SEM, SU8000, Hitachi, USA). Mg materials treated with 200 µg/ml collagen solution were used for elemental analysis. Energy dispersive X-ray spectroscopy (EDS, Qantax200, Bruker, USA) element mapping was performed with high voltage of 15 kV and probe current of 15 mA. Quantitative spectrum analysis was carried out after the spectra acquisition.

### Effect of pH

The pH of DPBS (7.49) solutions were adjusted to 7, 9, and 11 by 1 M NaOH (Sigma-Aldrich, USA) and 1 M HCl (Sigma-Aldrich, USA). Stock collagen solution was diluted by DPBS solutions with different pH to final concentration of 200 µg/ml. 50 µl final collagen solutions were spread on Mg and AZ31 surface (polished up to 1,200 grit SiC paper) for 2 hours of assembly, then followed by dehydration using ethanol gradient. The morphology of collagen was imaged by SEM.

### Effect of assembly time

50 µl of 200 µg/ml DPBS diluted collagen solution (pH of 7) was spread on Mg and AZ31 surface and allowed to assemble for 4 h and 8 h. Then samples were dehydrated with ethanol gradient. The morphology of collagen was imaged by SEM.

### Effect of surface roughness

Mg and AZ31 were divided into 3 groups and polished up to 180, 800 and 1,200 grit SiC paper, respectively. Materials polished up to 180 grit SiC paper were denoted as rough surface (RS); materials polished up to 800 grit SiC paper were denoted as semi-rough surface (SR); and materials polished up to 1,200 grit SiC paper were denoted as smooth surface (SS). Surface roughness was characterized by WYKO Optical Profiler (Veeco, USA). 50 µl 200 µg/ml DPBS diluted collagen solution (pH of 7) was spread on Mg and AZ31 surface and allowed to assemble for 2 h. The morphology of collagen fibril was imaged by SEM.

### Collagen dynamic adsorption

RS, SR and SS materials were soaked into 1 ml 60 µg/ml diluted collagen solution (pH of 7) with only one side exposed to the solution. The amount of absorbed collagen was quantified by Sirius Red Assay (Abacam, USA) according the method described previously with minor modification [33], [34]. In brief, materials were soaked into collagen solution for 0.5 h, 1 h, 2 h, 4 h, and 8 h, respectively. At each time point, samples were removed and washed with DPBS for 3 times. Then the unattached collagen in solution was transferred to a new tube followed by incubation with Sirius Red for 1 hour. The solutions were centrifuged at 8,000 g for 15 min and the dye was eluted by 0.1 M NaOH. The absorbance was measured at 540 nm by 10S UV-Vis Spectrometer (Thermo, USA). Standard curve of a series of collagen solution (7.5, 15, 22.5, 30, 37.5, 45, 52.5, and 60 µg/ml) was obtained as the same procedures. Linear regression was performed in Prism 5 (GraphPad, USA). The attached collagen was calculated by subtraction of initial collagen by the collagen remained in the solution.

### Cell attachment

Mouse osteoblasts (MC 3T3, ATCC, USA) was expanded in Minimum Essential Medium α (MEM, Life Technologies, USA) supplemented with 10% fetal bovine serum (Sciencell, USA), 100 U/ml penicillin (Sciencell, USA) and 100 µg/ml streptomycin (Sciencell, USA) in humidified incubator (Heracell 150 I, Thermo, USA) with 5% CO_2_ as previously described [35]. 50 µl 200 µg/ml collagen solution (pH of 7) was allowed to self-assemble on Mg and AZ31 with RS, SR, and SS for 2 h in a 24-well culture plate (BD Bioscience, USA). Then these materials were gently rinsed by DPBS for 3 times. 50 µl cell solution with density of 10,000 cell/ml was dipped onto the surface of collagen treated materials. Cells were allowed to attach for 30 min and then samples were washed gently with DPBS for 3 times. After 4 hours, cells were fixed with 4% paraformaldehyde (Boston Bioproducts, USA) followed by ethanol gradient dehydration for 10 minutes. Samples were coated (E5400, Polaron Instruments, USA) with gold nanoparticles for 2 min, and imaged by SEM.

### Cell proliferation

Mg and AZ31 with different surface roughness treated by collagen as described above were used to test cell proliferation. MC 3T3 Cells were seeded onto the collagen coated material surface with density of 10,000 cell/ml in a 24-well culture plate. At 1^st^, 4^th^, and 7^th^ day, cell culture media were changed and cells were stained by Live/Dead kit (Invitrogen, USA). Culture media were centrifuged (Biofuge Stratos, Thermo, USA) at 8,000 g for 10 min and pH was measured by a pH meter (Eutech, USA). The fluorescent images were taken by a digital inverted light microscope (EVOS, Advanced Microscopy, USA).

### Mg^2+^ concentration measurement

Mg^2+^ concentration was measured by xylidyl blue magnesium kit (Pointe Scientific, USA) as previously described [36]. In brief, 10 µl aliquot of test solution was added to 1.5 ml final xylidyl solution (0.1 mM xylidyl blue, 0.13 mM EGTA, 1.4 M DMSO, 0.02% potassium cyanide) and incubated for 10 min at room temperature. The absorbance of the mixtures was measured at 520 nm by a UV-Vis Spectrometer (Thermo, USA). Standard curve was obtained by using gradient MgCl_2_ solution. Linear regression was performed in Prism 5 (GraphPad, USA). Mg^2+^ concentration was determined by the standard curve.

### Statistics Analysis

Data was expressed as Mean ± SD in all figures unless otherwise specified. Statistical analysis was performed in Prism 5 software (GraphPad, USA). Unpaired two-tailed *t* test was used to compare difference between two groups. Multiple comparisons were performed by using one-way ANOVA followed by post hoc analysis. Two-way ANOVA was used to evaluate the effect of two independent factors. It is considered significantly different statistically if the *P*<0.05.

## Results

### Effect of collagen concentration

**Figure 1:**
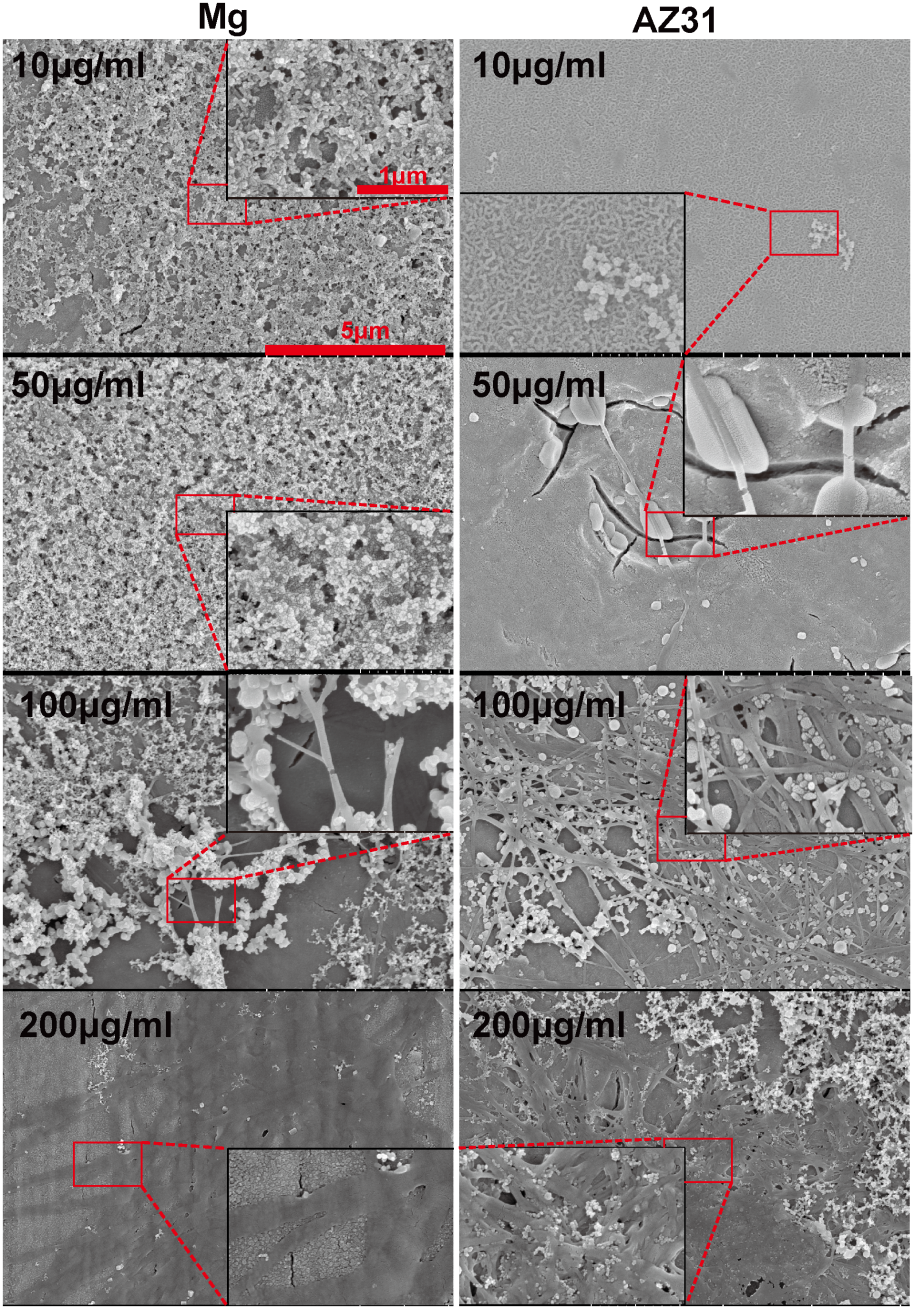
Representative SEM images of collagen self-assembly on Mg (left) and AZ31 (right). The concentration of collagen in each group is shown on the top left corner of each image.

We first explored collagen self-assembly with different concentrations of collagen monomers on SS of Mg and AZ31 at neutral pH (Fig. 1). On pure Mg, collagen monomers agglomerated into a non-uniform structure and no long fibril was observed when the concentration was lower than 50 µg/ml. Some spherical particles were present on the surface of pure Mg in both 10 µg/ml and 50 µg/ml groups. When the collagen concentration reached to 100 µg/ml, a few fibrils started to appear. The structure changed from thin fibrils to wide bands as the initial concentration increased to 200 µg/ml. On AZ31 surface, long fibril started to appear as the collagen concentration reached to 50 µg/ml. In addition, sparsely dispersed woven structure composed of collagen fibers (fibril bundles) were observed in the 100 µg/ml group. Multiple-layer network structure with collagen ribbon of 100 nm width was the predominant structure in the 200 µg/ml group. Spherical particles in different sizes were also present in all the groups.

**Figure 2:**
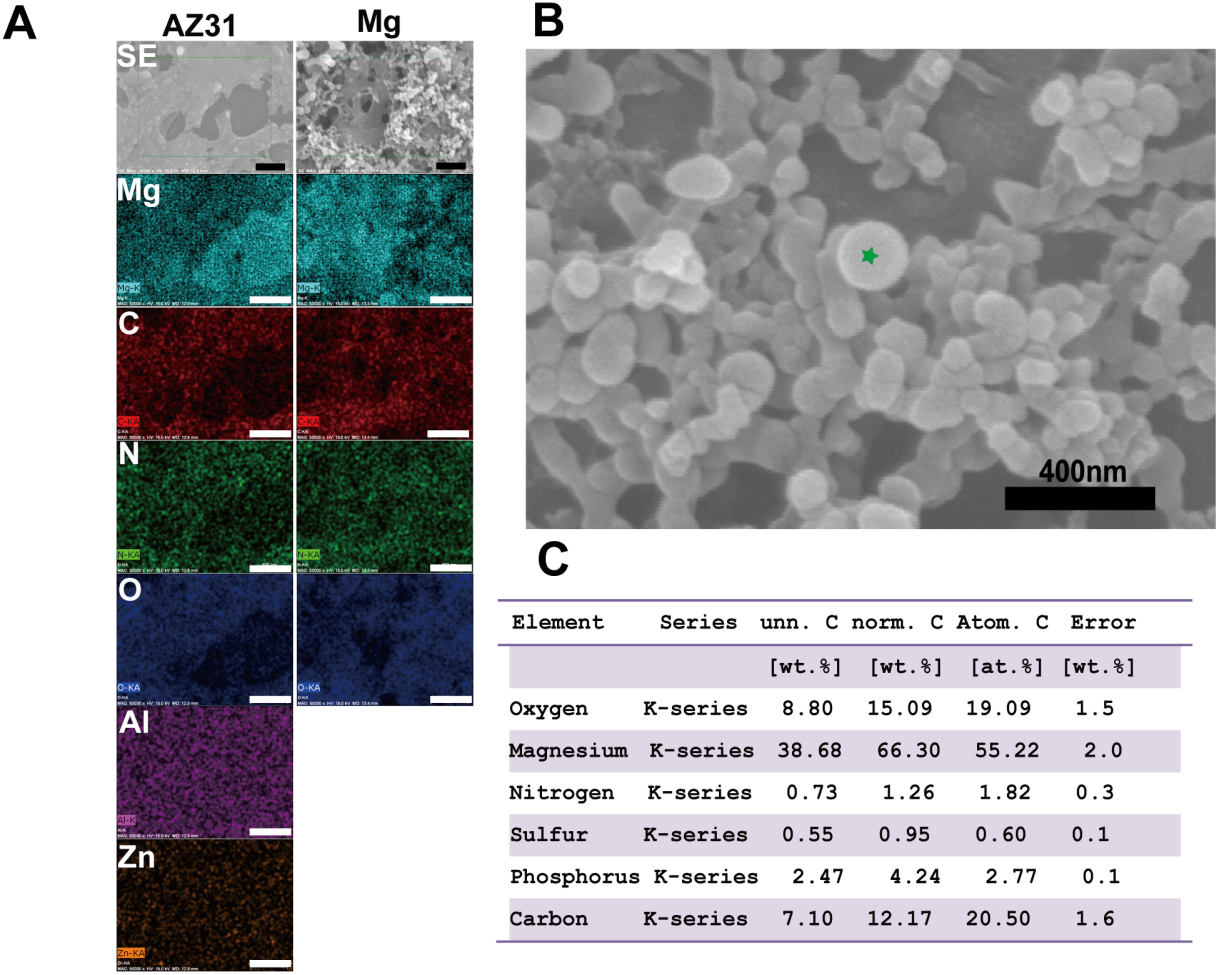
EDS analysis of collagen fibril and spherical particles on AZ31 and Mg. The corresponding element is shown on the top left corner. Scale bar is 400 nm. (A) EDS element mapping, (B) High magnification SEM image of nanoparticles attached on collagen fibril, (C) Elemental composition of a nanoparticle (green star in B) by EDS point analysis.

Next, we examined the chemical composition distribution of the fibril structure and the spherical particles by EDS (Fig. 2). Carbon (C), nitrogen (N) and oxygen (O) were mainly from the collagen fibrils structures on pure Mg and AZ31, which further demonstrated that they are not degradation products but collagen fibrils. In pure Mg group, less Mg was detected in some spherical particle dense zones. Al and Zn were evenly distributed on the collagen layer. A higher magnification image of the spherical particles (Fig. 2B) shows that the size of those particles is about one hundred nanometer. Their chemical composition is summarized in Fig. 2C.

### Effect of pH

**Figure 3:**
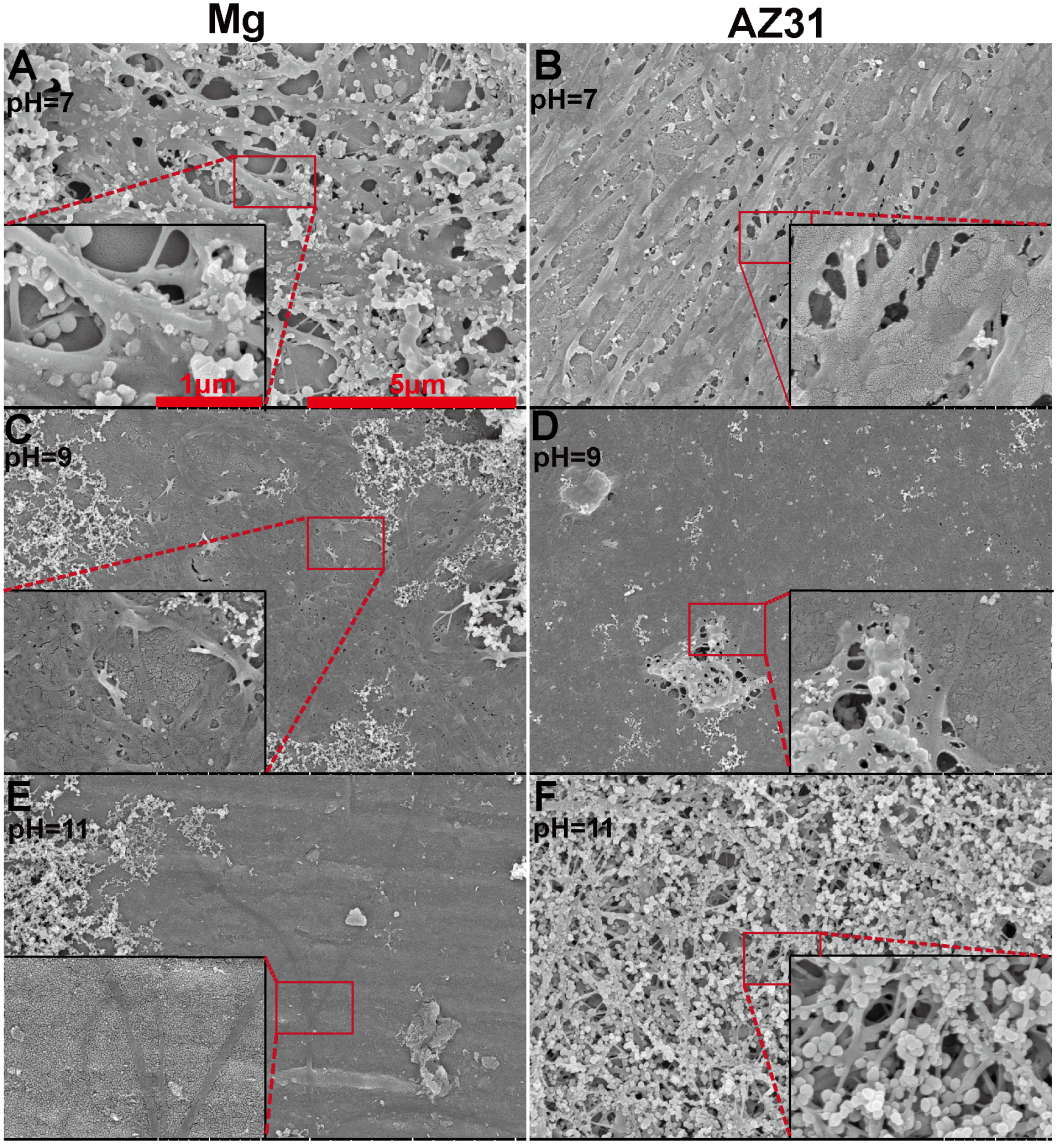
Representative SEM images of 200 µg/ml collagen self-assembly in DPBS with different pH values (A–B: 7, C–D: 9, E–F: 11) on Mg surface (A, C, E) and AZ31 (B, D, F).

Effect of pH on collagen assembly was also investigated. SEM images of collagen self-assembly under different pH on SS materials for 2 h are shown in Fig. 3. At pH 7, collagen ribbons with width ∼100 nm conjugated with other fibrils, forming a multiple layer network structure on pure Mg. A few nanofibril side chains connecting adjacent long collagen ribbon were also seen. On AZ31, some parallel collagen ribbons were connected with adjacent collagen ribbons by smaller branches and others merged with their proximal collagen, forming a uniform sheet. At pH 9, more collagen ribbons wove together spreading on Mg surface. In addition, some bare areas and crevices were observable. The whole surface of AZ31 was almost covered by a collage layer with some small holes. Few long collagen fibrils were present on pure Mg when pH rose to 11. On AZ31, thin fibrils randomly crossed with others, resulting in a network structure. A lot of nano-spherical particles attached to the collagen fibrils.

### Effect of reaction time

**Figure 4:**
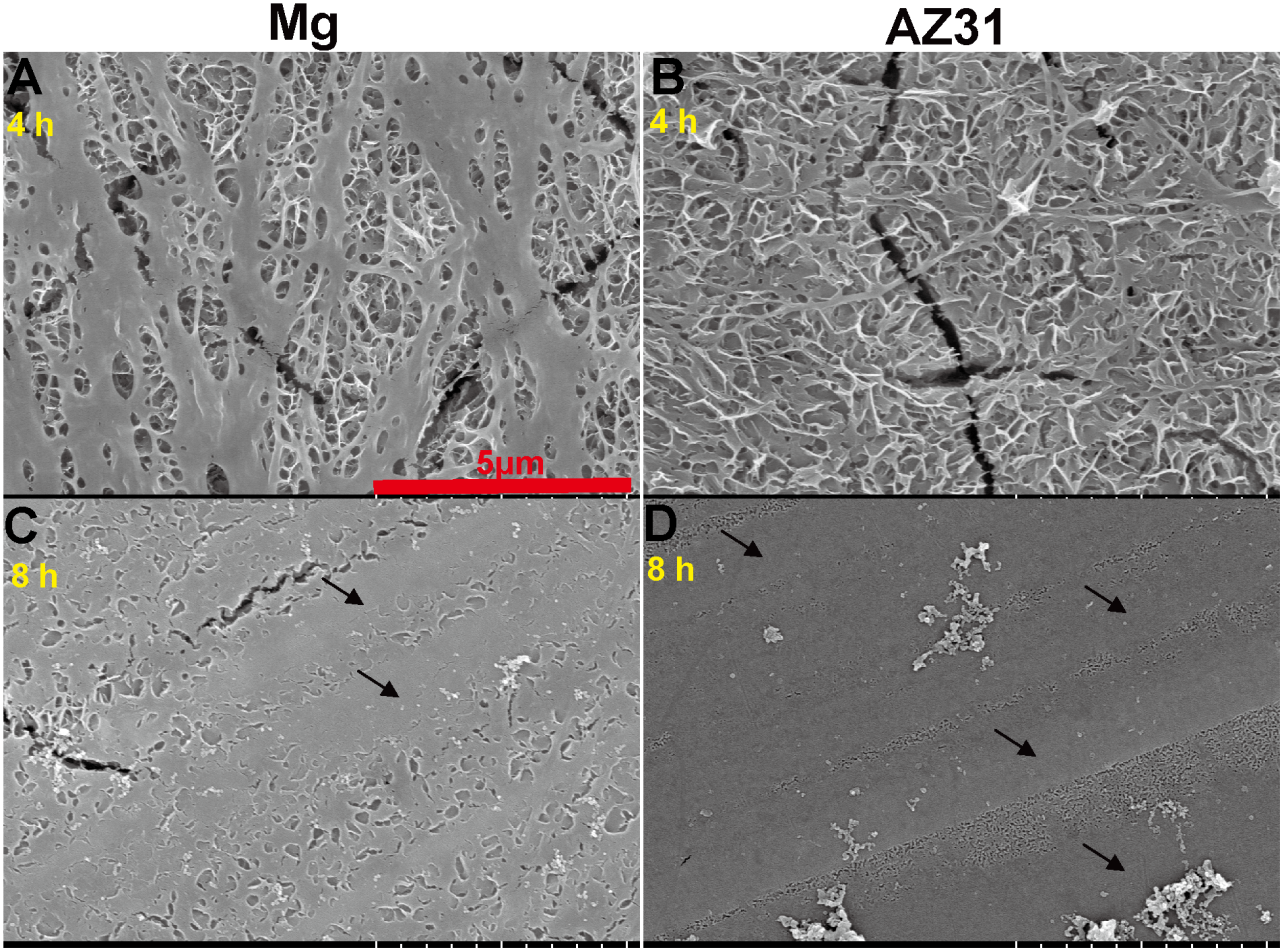
Representative SEM images of 200 µg/ml collagen self-assembly on Mg (A, C) and AZ31 (B, D) for 4 h (A, B) and 8 h (C, D). Arrows indicate the large collagen band.

Fig. 4 shows the structures of collagen assembling for different time periods on SS materials at neutral pH. After 4 hours assembling, a cancellous underneath layer was covered by some long collagen ribbons on pure Mg. Similar cancellous structure was found on AZ31, but less thin collagen fibrils could be observed on the top. When the assembly time reached 8 hours, micrometer-wide fibers were present on both pure Mg and AZ31. On pure Mg, a lot of small lamellar sheets were observed between two thick fibers.

### Effect of surface roughness

**Figure 5:**
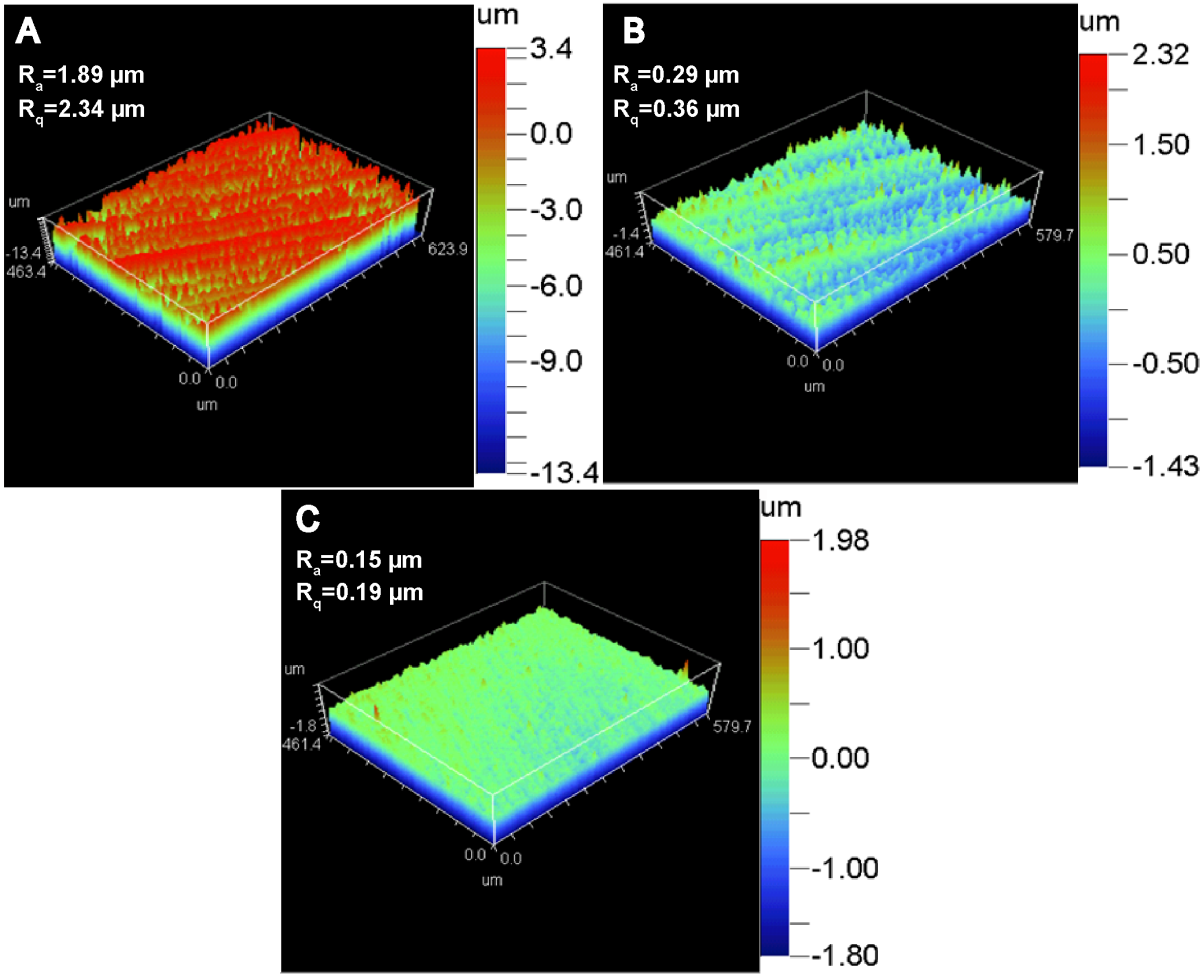
3-D surface topography of Mg sample polished by different SiC paper (A: 180 grit; B: 800 grit; C: 1200 grit). The arithmetic average of surface roughness (R_a_) and root mean square (R_q_) calculated from 4 different samples are shown on the top left corner.

**Figure 6:**
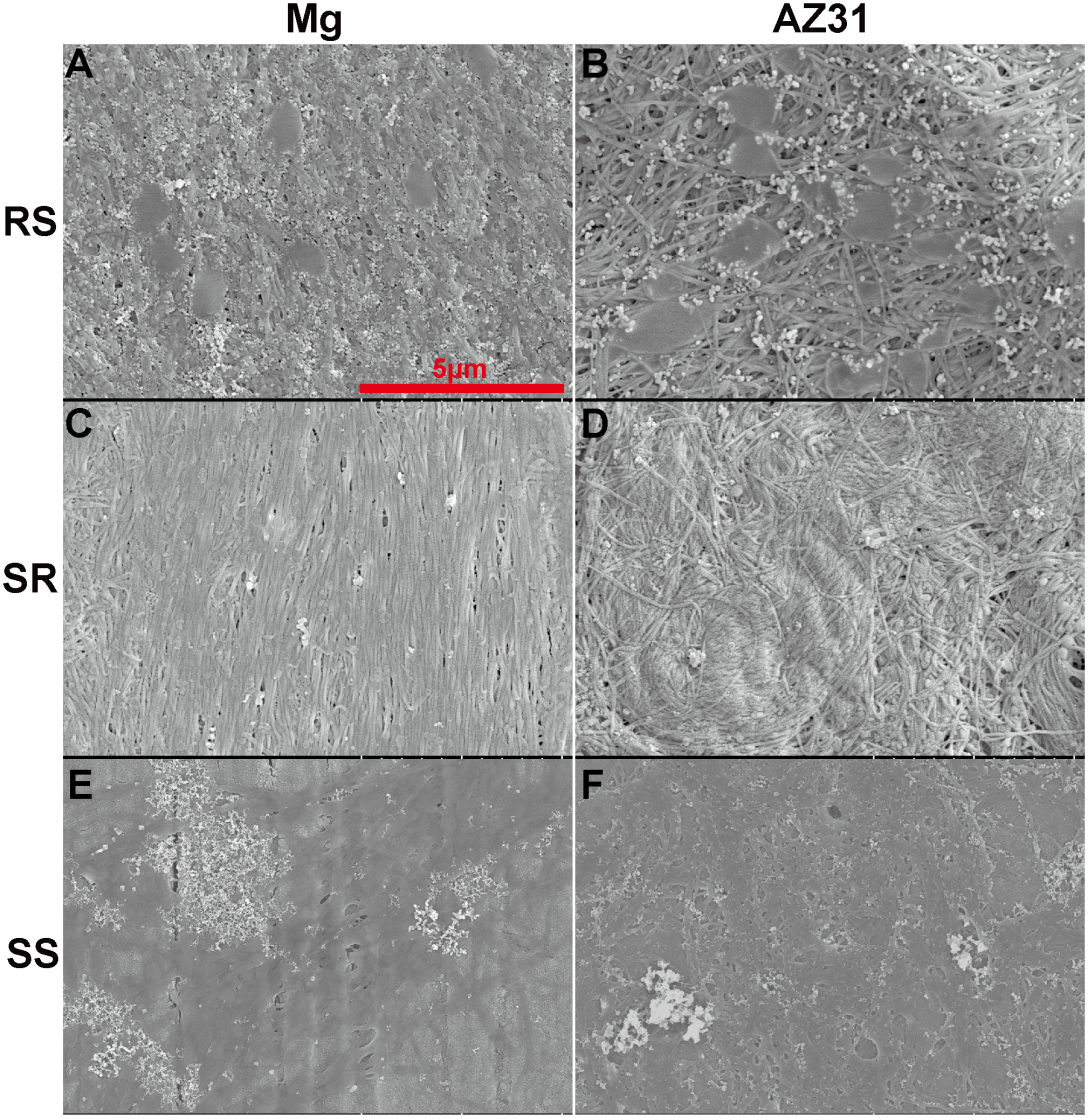
Representative SEM images of 200 µg/ml collagen self-assembly on Mg (A, C, E) and AZ31 (B, D, F) with different surface roughness (A–B: RS; C–D: SR; E–F: SS). RS: rough surface; SR: semi-rough surface; SS: smooth surface.

Representative 3-D topographical images of materials after polished by different SiC paper are shown in Fig. 5. The average roughness (R_a_) and the corresponding root mean squared roughness (R_q_) were also calculated (Fig. 5). Fig. 6 shows the collagen self-assembly on the samples with different surface roughness. On the RS Mg materials, long collagen fibers interwove with each other resulting in a compact woven layer with a few fish-like scales on the top. The woven layer on RS AZ31 was less denser compared with that on RS Mg. Larger and more fish-like scales structures could be seen on the RS AZ31. For both AZ31 and pure Mg with SR, collagen fibers aligned parallelly to each other at some places while randomly intertwined at other places. On SS of both materials, collagen bands were similar as described previously (Fig. 1).

### Collagen assembly quantification

**Figure 7:**
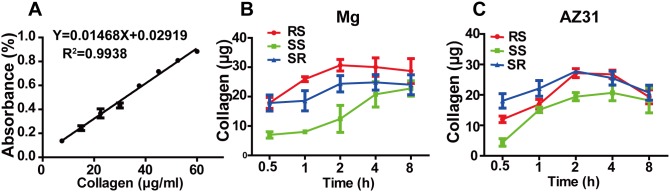
Collagen adsorption quantification. Standard curve for quantification of collagen by Picro-sirius red stain kit (A). The amount of collagen absorbed on Mg with different surface roughness at 0.5, 1, 2, 4, and 8 h (B). The amount of collagen absorbed on AZ31 with different surface roughness at 0.5, 1, 2, 4, and 8 h (C).

We also quantified the amount of collagen on the material surface during its assembly. A standard curve for quantification of collagen content was established (Fig. 7A). The optical absorbance at 540 nm versus collagen content displayed reasonable linearity within the range from 5 to 60 µg/ml. Collagen dynamic adsorption curves on Mg and AZ31 surfaces are shown in Fig. 7B and Fig. 7C. For both AZ31 and pure Mg, less collagen was able to be absorbed onto the SS materials at the initial phase (0.5 h) compared with RS and SR materials. The amount of attached collagen increased as the adsorption time increased and reached equilibrium state after 4 h for all three groups of AZ31. Collagen on the pure Mg with SS kept increasing slowly from 0.5 to 2 h. In comparison, collagen on pure Mg with RS peaked at 2 h and then started to drop. Among all the groups, the highest amount of absorbed collagen was 30.69±1. 96 µg on RS Mg at 2 h. Two-way ANOVA analysis revealed that both time and surface roughness had significant effect on collagen adsorption on pure Mg.

### Bone cell attachment and proliferation

**Figure 8:**
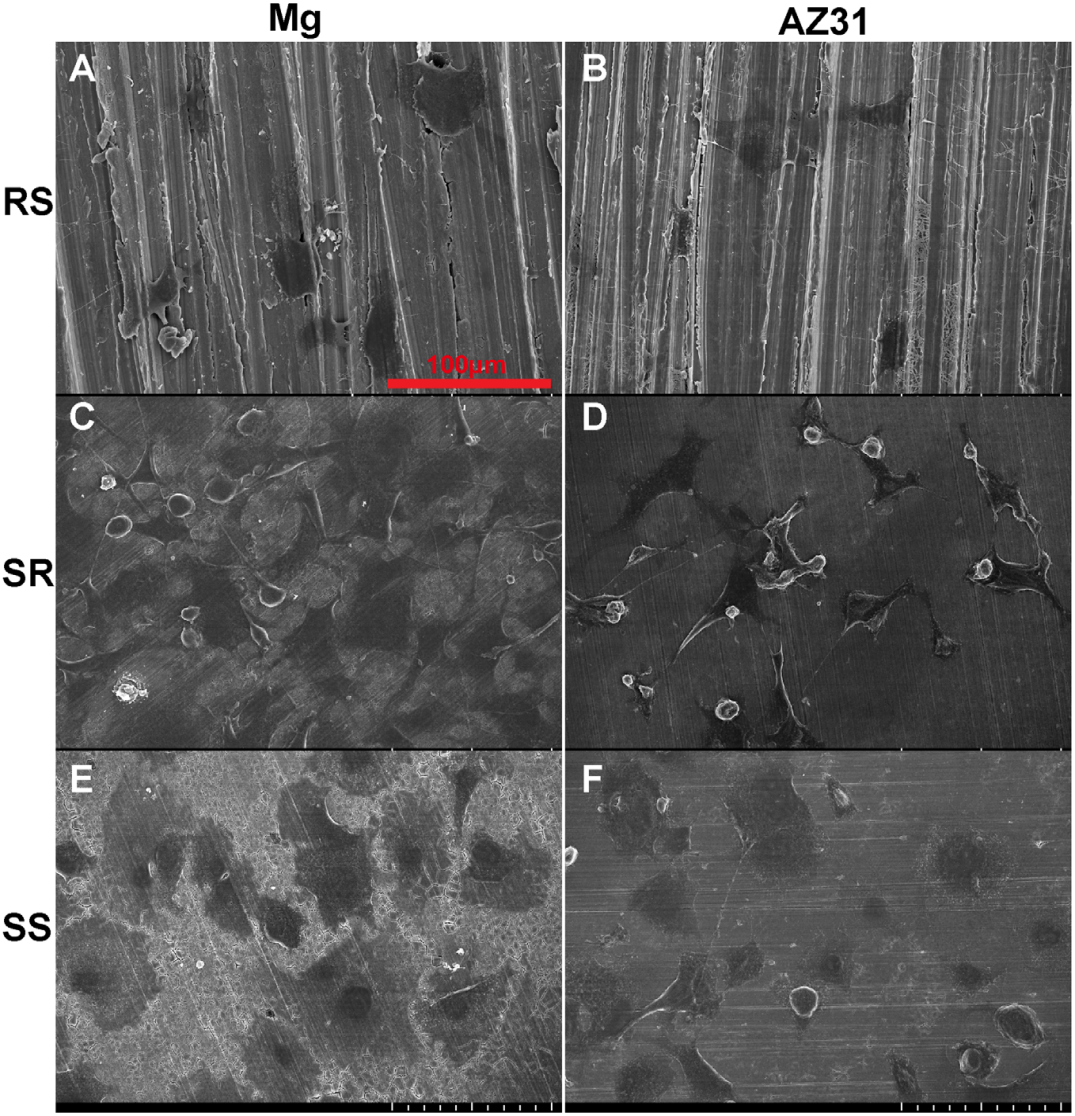
Representative SEM images of MC 3T3 cell attachment on collagen self-assembled at Mg (A, C, E) and AZ31 (B, D, F) with different surface roughness (A–B: RS; C–D: SR; E–F: SS).

After Mg and AZ31 with different surface roughness were treated by 200 µg/ml collagen solution for 2 h, the materials were used to test subsequent bone cell attachment (Fig. 8). SEM images showed that both round cells and cells with filopodia were observed on RS AZ31 and pure Mg. On SR materials, most cells were well attached with flattened morphology and a few fibroblast-like cells with webbing could also be seen. In addition, super long filopodia from some cells span over a large distance and reached the edge of other cells or an empty area. On the SS materials, most cells were well flattened with very large cell surface area. Cell density on SR and SS was significantly higher than that on RS.

**Figure 9:**
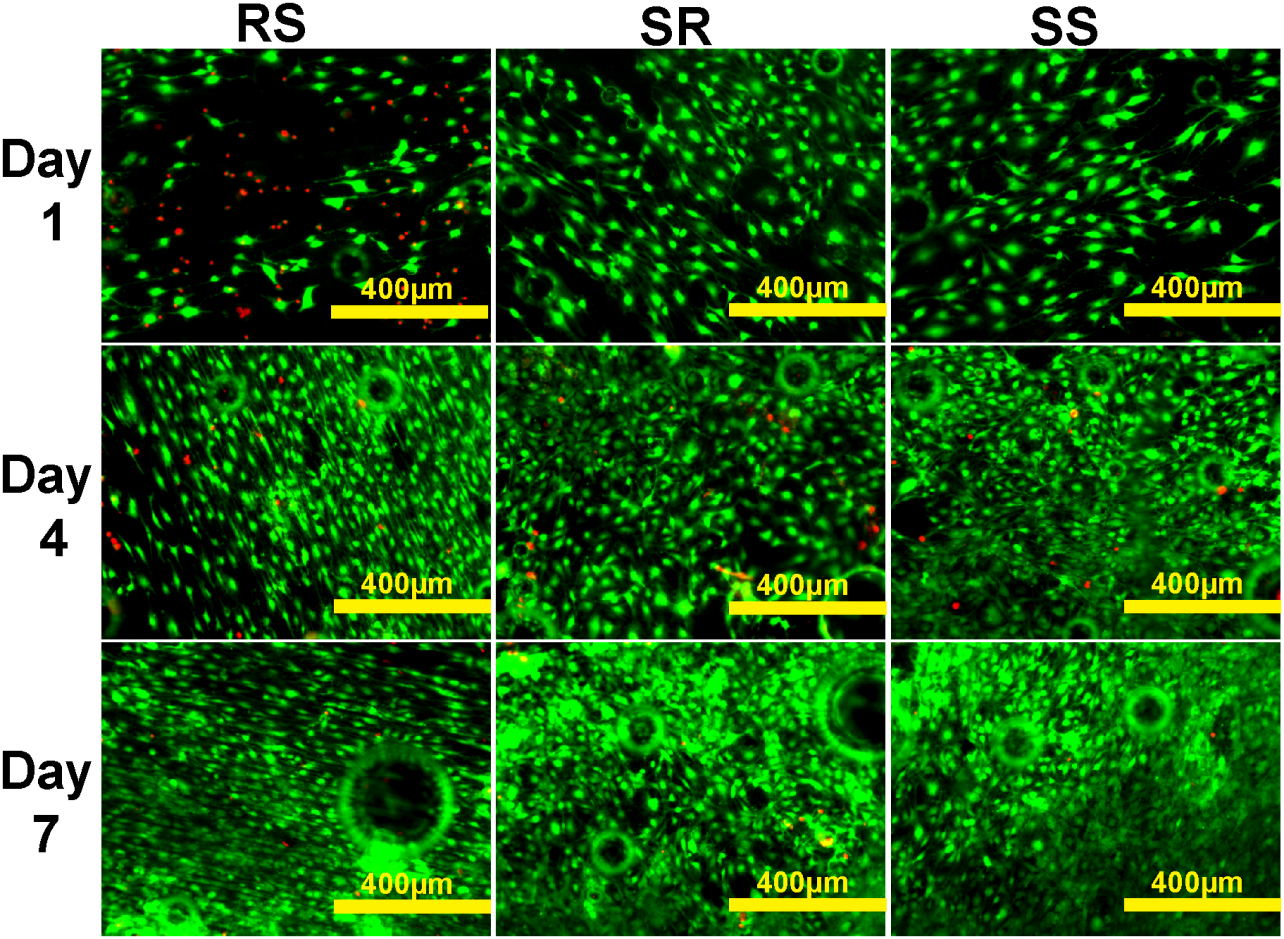
Representative fluorescent images of MC 3T3 cells growing on collagen self-assembled at AZ31 with different surface roughness for 1, 4 and 7 d. Live cells are in green color and dead cells are in red color.

**Figure 10:**
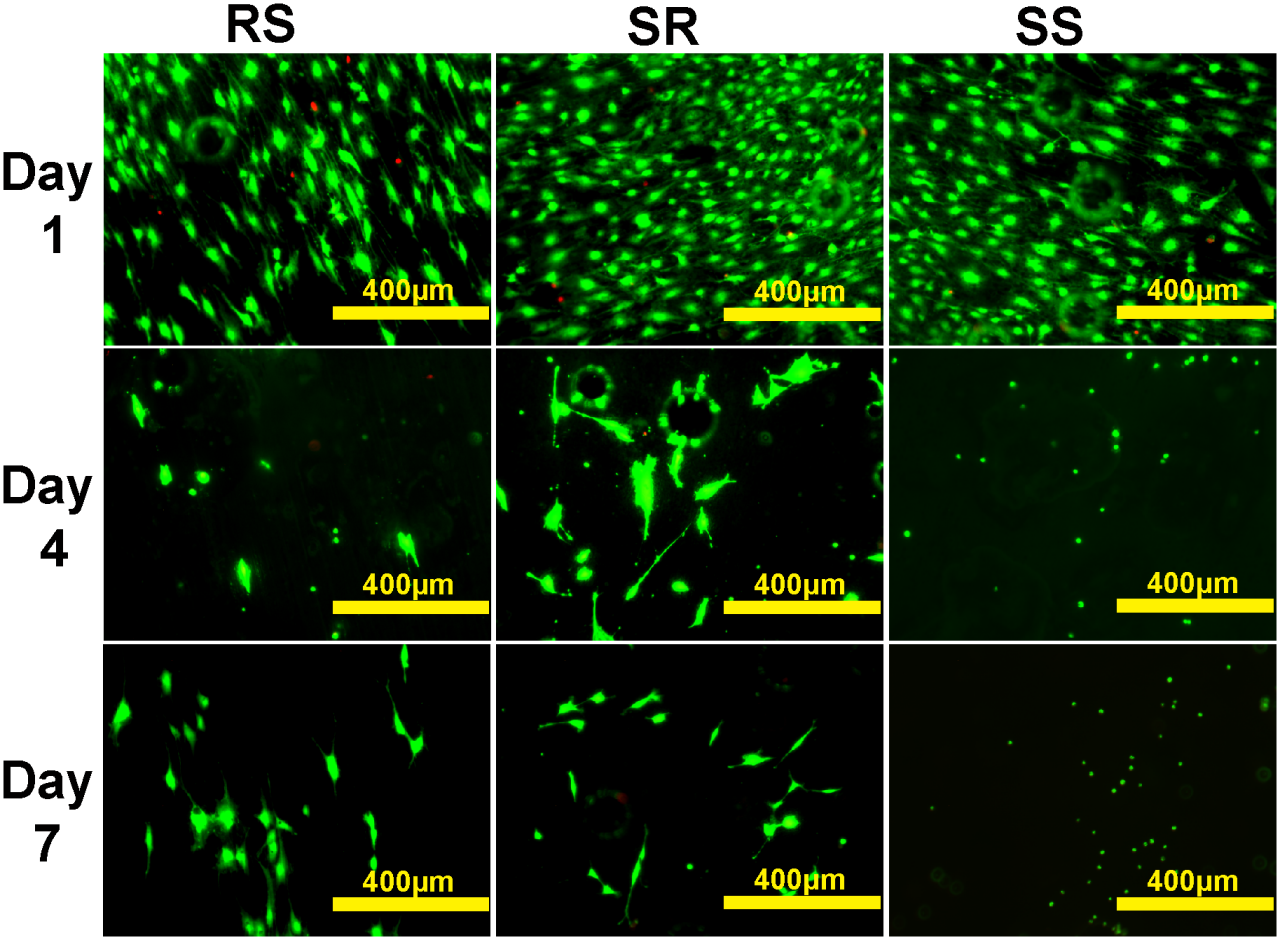
Representative fluorescent image of MC 3T3 cells growing on collagen self-assembled at Mg with different surface roughness for 1, 4 and 7 d. Live cells are in green color and dead cells are in red color.

**Figure 11:**
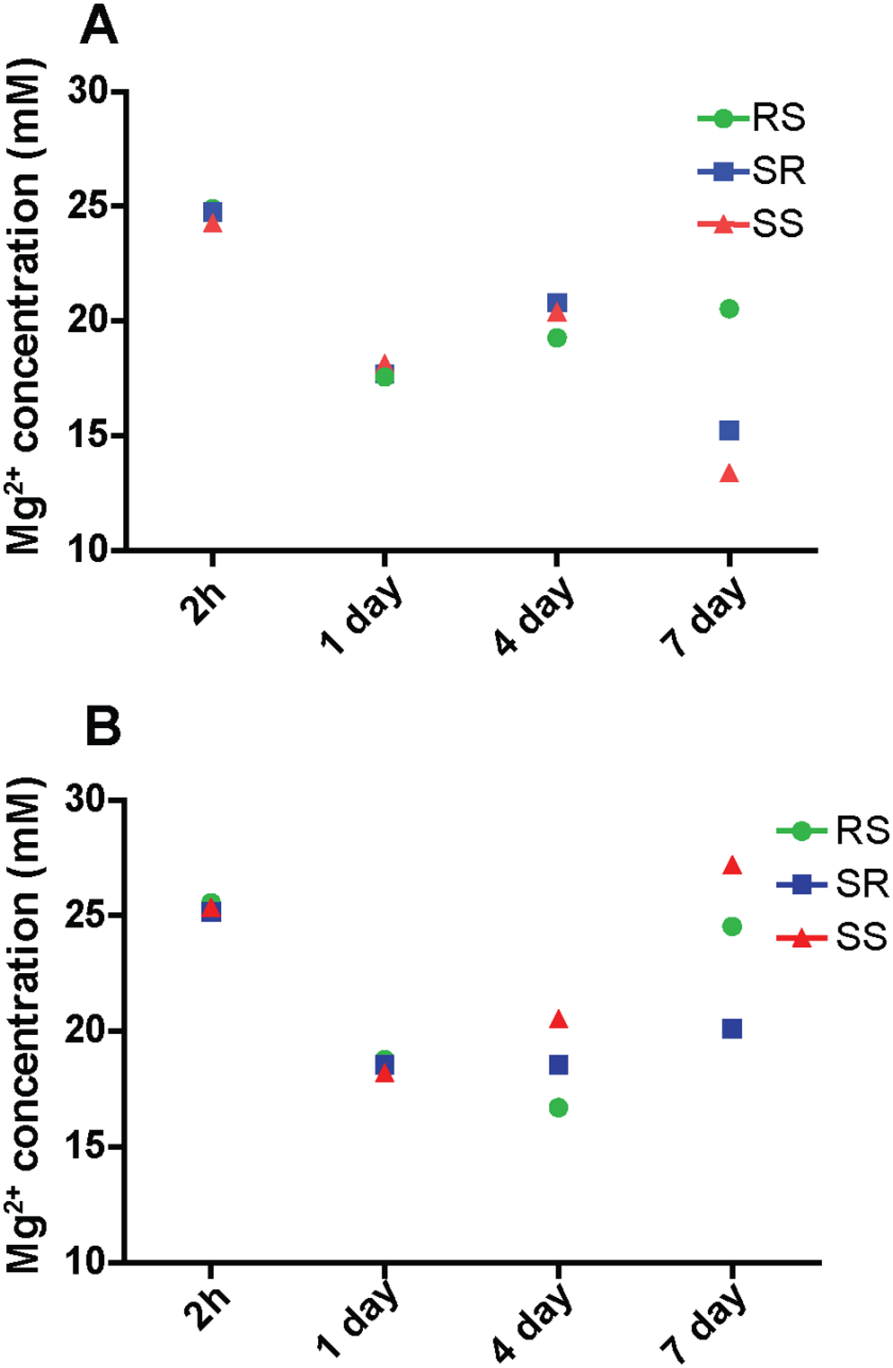
Mg^2+^ concentration after the materials (A-AZ31; B-Mg) were incubated with collagen solution for 2 h and cocultured with cells for 1, 4 and 7 days. Error bars were omitted for clarity purpose.

Fluorescent live/dead cell analysis was then performed to examine the bone cell proliferation. MC 3T3 cells on AZ31 and Mg treated with 200 µg/ml collagen solution for 2 h are shown in Fig. 9 and Fig. 10, respectively. Cells displayed healthy morphology in all the groups after one day. Cell densities on SR and SS AZ31 were significantly higher than that on RS AZ31 (Fig. 9). Some dead cells were visible after first day on RS AZ31. Small air bubbles were present in all the materials. After three days’ incubation, cell density on all the AZ31 materials increased. On the RS AZ31, cells elongated at the same direction. After seven days, cell density further increased and multiple layers of cells could be seen in all the three groups. Most of the cells on the RS group still aligned in the same direction. Larger hydrogen gas bubbles emerged in all groups. On pure Mg, cell densities significantly decreased at 4^th^ and 7^th^ day. At the first day, cells showed similar uniform elongation on the RS pure Mg. However, normal spreading cells could barely be observed on the SS Mg at 4^th^ and 7^th^ day. Table 1 summarizes the pH value of cell culture media during the culture. In both AZ31 and Mg groups, the materials with RS showed significantly higher pH change than the materials with SR and SS after the first day. Mg^2+^ concentration (Fig. 11) after collagen was incubated with Mg and AZ31 of different surface roughness for 2 h was all around 25 mM. In contrast, Mg^2+^ concentration in the cell culture media was significantly lower than that in the collagen solution for the AZ31 group from 1 day to 7 day.

**Table 1:** The pH of the cell culture media after incubated with cells on materials.

Material		pH		
		1 day	4 day	7 day
AZ31	RS	7.93±0.05	7.88±0.03	7.94±0.03
	SR	7.70±0.08	7.92±0.03	7.85±0.05
	SS	7.75±0.08	7.91±0.06	7.76±0.07
Mg	RS	8.30±0.14	7.95±0.03	7.96±0.03
	SR	8.01±0.06	7.92±0.03	7.92±0.03
	SS	8.13±0.06	7.95±0.03	7.87±0.03

## Discussion

Collagen, the ubiquitous ECM component, is a large family of triple-helical proteins. So far, around 28 types of collagen have been identified. Among them, type I collagen is the most abundant type, which forms the backbone of ECM in a lot of tissues such as bone, dermis, and tendon. 90% of the organic weight of bone is made up of type I collagen [37]. Type I collagen triple helix is composed of heterotrimer of two identical α1(I) chains and one α2(I) chain. Procollagen molecule is synthesized inside cells followed by post-translational modifications and then assemblies into triple helix procollagen with diameter of 1.5 nm and length of 300 nm [37]. Then it is secreted to extracellular space by secretory vesicles and further processed by different proteinases. In vitro, collagen fibrils are formed by self-assembly into cross-striated fibrils with the characteristic D-period of 67 nm [38]. In natural bone tissues, collagen fibrils are the scaffold for biomineralization. It is believed that collagen molecules are secreted as amorphous and non-crystalline forms and then transformed into crystalline forms gradually [39]. Mg-based alloys have promising future for orthopedic applications with respect to their mechanical properties, degradation properties, and biocompatibility. While the exact mechanism of collagen fibril formation on Mg surface in vivo remains unknown, in vitro self-assembly model established in this work provides a simple and alternative way to study how Mg materials interact with collagen molecules.

Collagen fibril formation on mica surface involves the adsorption of collagen molecules, surface diffusion, nucleation of fibrils and fibril elongation [40], [41]. A lot of studies have shown that collagen could self-assembly into axially aligned fibrils with D-period similar to native bone tissues [42], [43]. However, the assembly of collagen on mica surface could be different from that on Mg-alloy surface due to their distinct surface characteristics and electrostatics. Once in contact with body fluid, the metal elements in Mg materials will be oxidized into metal cations followed by the formation of a layer of metal hydroxide [44]. Metal ion would be released to the fluid during the degradation process and biomacromolecules such as protein, proteoglycan, and glycoprotein can be absorbed to the metal hydroxide layer [44]. It is interesting that for both pure Mg and AZ31 with different surface roughness, Mg ion released to the collagen solution after 2 h incubation didn’t show significant difference. This is most likely due to the small total volume of solution (50 µl) and Mg ion was already saturated in the solution. At neutral pH, this metal hydrochloride layer is beneficial for collagen molecule adsorption since collagen molecule is positively charged. The absence of large fibril at low concentration of collagen monomer is most likely caused by the decreased chance for fibril nucleation. The concentration of collagen monomer can also affect the fibril growth rate and single fibrils grow independent from each other until they fuse with adjacent fibrils [40]. In addition, it was shown by Wang et al. that at low concentration collagen monomers form agglomerates in solution containing excessive Mg ions [45]. Similar agglomerates structure was also observed here on pure Mg and AZ31 surface at low collagen concentration. This might be caused by the high Mg^2+^/collagen ratio and excessive Mg^2+^ could bind to collagen side chain leading to the increase of protein hydrophobicity and the dehydration of collagen [46]. Besides the release of metal ions, pH change accompanying the degradation process is another important factor that could affect collagen assembly. In the absence of other electrolytes, the isoelectric point (pI) of collagen is around 9.3 [47]. When pH approximates pI, the surface charge of collagen monomers is decreased resulting in minimized electrostatic repulsion and better fibril assembly. This is supported by our data (Fig. 3) where collagen fibrils loosely aligned at pH of 7 while they formed a dense layer of sheet at pH 9. As pH increased to 11, negatively charged collagen monomers could inhibit the nucleation of collagen fibril as well as the further attachment to Mg hydroxide layer. With the increase of incubation time to 8 h, small collagen fibrils could merge with adjacent fibrils forming thicker fibers (Fig. 4C and Fig. 4D). It is interesting to see that almost in all experiments spherical particles with different sizes were attached to collagen fibrils regardless of the diameters of collagen fibrils. The shape and size of those particles are very similar to the mineral nucleation reported by Ferreia et al [48]. However, from the EDS elemental analysis (Fig. 2), the particle structures are most likely magnesium compound instead of bone mineral.

It is well documented that implant surface roughness alters osteoblast proliferation, differentiation, and extracellular matrix production [16]. Mendonca et al. showed that rough surface topography can stimulate collagen biosynthesis and accumulation on titanium [17]. Mg materials with RS have relative larger surface area that increases the chance of collagen molecules adsorption. This is probably why the amount of collagen absorbed on the RS and SR materials was significantly higher than that on materials with SS. Also, surface energy could affect collagen adsorption and structural rearrangement. It is noticeable that the amount of absorbed collagen decreased at 8 h on the materials with RS and SR. This phenomenon is most likely caused by severer pitting corrosion on rougher surface compared with smoother surface [49], [50]. In addition, surface roughness not only affected the amount of collagen absorbed but also the structure of the fibrils (Fig. 6). The slightly morphological difference of collagen fibrils on Mg and AZ31 is likely caused by the presence of Zn^2+^ and Al^3+^, the AZ31 degradation products [47]. Therefore, ion release rate, local pH change, hydrogen gas formation, surface energy and surface electrostatic properties can all affect the final fibril structure.

We further studied how those materials affect cell attachment and proliferation. The better cell attachment on the materials with SS (Fig. 8) is consistent with previous studies [51]. On AZ31 material, a lot of dead cells could be observed on the RS materials after the first day (Fig. 9). This is most likely due to the failure of cell attachment or hampered cell attachment on the RS where cells could only anchor themselves at reduced area caused by the existence of the grooves and ridges. The grooves and ridges showed contact guidance effect on cell alignment. It was demonstrated before that the tip of filopodia most likely attaches to the top of the ridges [52]. During cell migration, it would be much easier for cell to move the tip of the adhesion along the ridge than to move the tip of the adhesion perpendicular to the direction of ridges. That may be the reason why cells on the rough surface materials all aligned parallel to the direction of ridges. Cells showed similar proliferation results on AZ31 with different surface roughness indicating that surface roughness and collagen structure won’t affect cell proliferation. However, cells didn’t show similar proliferation result on pure Mg at 4^th^ day and 7^th^ day. Cell density significantly decreased at the Mg with RS and SR. Healthy spreading cells could hardly be found on the SS of pure Mg materials. At body temperature, melting time for human type I collagen is around several days [53]. Compared with AZ31, the relative faster degradation rate of pure Mg could lead to higher concentration of degradation products and higher pH in solution, which might accelerate dissociation of attached collagen and cause decreased cell density. In addition, the thick collagen ribbon structure doesn’t resemble native collagen structure in bone. The collagen fibrils in Fig. 6C and Fig. 6D showed highly similarity with the demineralized circumferential lamellar bone [54]. Ideally, the preferable orthopedic implants should not only be able to stimulate bone cell growth but also to support the assembly of collagen monomer into native fibrils at the bone-implant interface.

This in vitro model was developed to mimic the in vivo interactions between collagen and the Mg implant at the interface. It provided useful information on the molecular mechanism of such an interaction that will influence the fate of the implant. It may also have some limitations. For example, different cell regulations and other protein interactions were neglected. Other types of bone cells and non-collagenous proteins also play important roles in collagen assembly [55]. Therefore, more future studies are needed to address these factors. Additionally, one interesting topic for next step could be to investigate how mineralization happens around the interfaces.

## Conclusion

In sum, collagen monomer can form different structures on Mg biomaterial depending on the initial collagen monomer concentration, pH in the solution, assembly time, electrolytes, and the material surface roughness. Only the initial collagen monomer concentration reaches a certain level, can long collagen fibrils form on Mg materials surface. The pH can change the electrostatic properties of collagen and final collagen structure. With the increase of collagen assembly time, small fibrils merge with adjacent fibrils forming thicker fibers. Materials with rough surface show stronger collagen adsorption ability. Initial cell attachment on RS of materials decreased independent of the composition of materials. AZ31 surface roughness and collagen structure did not affect cell proliferation for up to 7 days. Materials with high degradation rate may not change collagen assembly structure but can affect MC 3T3 cell proliferation. More studies on the combined effects of those factors such as pH, material surface roughness, assembly time and electrolytes on collagen assembly, mineralization and bone cell interaction are needed in the future. By controlling those factors, one can not only change the amount of absorbed collagen on Mg material surface but also the collagen fibril structure.

## References

[1] Gu X, Xie X, Li N, Zheng Y, Qin L (2012) In vitro and in vivo studies on a Mg–Sr binary alloy system developed as a new kind of biodegradable metal. Acta Biomater 8: 2360–2374. 22387336 10.1016/j.actbio.2012.02.018

[2] Li Z, Gu X, Lou S, Zheng Y (2008) The development of binary Mg–Ca alloys for use as biodegradable materials within bone. Biomaterials 29: 1329–1344. 18191191 10.1016/j.biomaterials.2007.12.021

[3] Xu L, Yu G, Zhang E, Pan F, Yang K (2007) In vivo corrosion behavior of Mg-Mn-Zn alloy for bone implant application. Journal of Biomedical Materials Research Part A 83: 703–711. 17549695 10.1002/jbm.a.31273

[4] Kim W, Kim W (2014) Fabrication of ultrafine-grained Mg–3Al–1Zn magnesium alloy sheets using a continuous high-ratio differential speed rolling technique. Materials Science and Engineering: A 594: 189–192.

[5] Hanada K, Matsuzaki K, Huang X, Chino Y (2013) Fabrication of Mg alloy tubes for biodegradable stent application. Materials Science and Engineering: C 33: 4746–4750. 24094183 10.1016/j.msec.2013.07.033

[6] Perez P, Onofre E, Cabeza S, Llorente I, del Valle JA, et al (2013) Corrosion behaviour of Mg-Zn-Y-Mischmetal alloys in phosphate buffer saline solution. Corrosion Science 69: 226–235.

[7] Ji DW, Liu CM, Chen ZY, Wang HH, Wang B (2013) Effects of Zn content on microstructures and mechanical properties of as cast Mg-Zn-Y-Zr alloys. Materials Science and Technology 29: 480–486.

[8] Bornapour M, Muja N, Shum-Tim D, Cerruti M, Pekguleryuz M (2013) Biocompatibility and biodegradability of Mg-Sr alloys: the formation of Sr-substituted hydroxyapatite. Acta Biomater 9: 5319–5330. 22871640 10.1016/j.actbio.2012.07.045

[9] Seitz JM, Eifler R, Stahl J, Kietzmann M, Bach FW (2012) Characterization of MgNd2 alloy for potential applications in bioresorbable implantable devices. Acta Biomaterialia 8: 3852–3864. 22676917 10.1016/j.actbio.2012.05.024

[10] Mao L, Yuan G, Wang S, Niu J, Wu G, et al (2012) A novel biodegradable Mg–Nd–Zn–Zr alloy with uniform corrosion behavior in artificial plasma. Materials Letters 88: 1–4.

[11] Xu S, Oh-Ishi K, Kamado S, Uchida F, Homma T, et al (2011) High-strength extruded Mg–Al–Ca–Mn alloy. Scripta Materialia 65: 269–272.

[12] Staiger MP, Pietak AM, Huadmai J, Dias G (2006) Magnesium and its alloys as orthopedic biomaterials: a review. Biomaterials 27: 1728–1734. 16246414 10.1016/j.biomaterials.2005.10.003

[13] Witte F, Kaese V, Haferkamp H, Switzer E, Meyer-Lindenberg A, et al (2005) In vivo corrosion of four magnesium alloys and the associated bone response. Biomaterials 26: 3557–3563. 15621246 10.1016/j.biomaterials.2004.09.049

[14] Choudhary L, Singh Raman R (2012) Magnesium alloys as body implants: Fracture mechanism under dynamic and static loadings in a physiological environment. Acta Biomater 8: 916–923. 22075121 10.1016/j.actbio.2011.10.031

[15] Zhao N, Zhu D (2013) Application of Mg–based alloys for cardiovascular stents. International Journal of Biomedical Engineering and Technology 12: 382–398.

[16] Martin J, Schwartz Z, Hummert T, Schraub D, Simpson J, et al (1995) Effect of titanium surface roughness on proliferation, differentiation, and protein synthesis of human osteoblast-like cells (MG63). Journal of biomedical materials research 29: 389–401. 7542245 10.1002/jbm.820290314

[17] Mendonça D, Miguez PA, Mendonça G, Yamauchi M, Aragão FJ, et al (2011) Titanium surface topography affects collagen biosynthesis of adherent cells. Bone 49: 463–472. 21549232 10.1016/j.bone.2011.04.019

[18] Nebe J, Lüthen F, Rychly J, Eisenbarth E, Müller M, et al.. (2007) Topology-Dependent Cellular Interactions. Metallic Biomaterial Interfaces: 215–248.

[19] Anselme K (2000) Osteoblast adhesion on biomaterials. Biomaterials 21: 667–681. 10711964 10.1016/s0142-9612(99)00242-2

[20] Boyan BD, Hummert TW, Dean DD, Schwartz Z (1996) Role of material surfaces in regulating bone and cartilage cell response. Biomaterials 17: 137–146. 8624390 10.1016/0142-9612(96)85758-9

[21] Nudelman F, Pieterse K, George A, Bomans PH, Friedrich H, et al (2010) The role of collagen in bone apatite formation in the presence of hydroxyapatite nucleation inhibitors. Nature materials 9: 1004–1009. 20972429 10.1038/nmat2875 PMC3084378

[22] Ambrose SH (1990) Preparation and Characterization of Bone and Tooth Collagen for Isotopic Analysis. Journal of Archaeological Science 17: 431–451.

[23] Bailey LKaAJ (1998) Collagen Cross-Links in Mineralizing Tissues: A Review of Their Chemistry, Function, and Clinical Relevance. Bone 22: 181–187. 9514209 10.1016/s8756-3282(97)00279-2

[24] Franz CM, Muller DJ (2011) Studying collagen self-assembly by time-lapse high-resolution atomic force microscopy. Atomic Force Microscopy in Biomedical Research: Humana Press. 97–107. 10.1007/978-1-61779-105-5_7 21660723

[25] Stephanopoulos N, Ortony JH, Stupp SI (2013) Self-assembly for the synthesis of functional biomaterials. Acta materialia 61: 912–930. 23457423 10.1016/j.actamat.2012.10.046 PMC3580867

[26] O’Leary LE, Fallas JA, Bakota EL, Kang MK, Hartgerink JD (2011) Multi-hierarchical self-assembly of a collagen mimetic peptide from triple helix to nanofibre and hydrogel. Nature chemistry 3: 821–828. 10.1038/nchem.1123 21941256

[27] Wang ZL, Yan YH, Wan T, Yang H (2013) Poly(L-lactic acid)/hydroxyapatite/collagen composite coatings on AZ31 magnesium alloy for biomedical application. Proc Inst Mech Eng H 227: 1094–1103. 23851659 10.1177/0954411913493845

[28] Sader MS, Martins VC, Gomez S, LeGeros RZ, Soares GA (2013) Production and in vitro characterization of 3D porous scaffolds made of magnesium carbonate apatite (MCA)/anionic collagen using a biomimetic approach. Mater Sci Eng C Mater Biol Appl 33: 4188–4196. 23910332 10.1016/j.msec.2013.06.006

[29] Ao H, Xie Y, Tan H, Yang S, Li K, et al (2013) Fabrication and in vitro evaluation of stable collagen/hyaluronic acid biomimetic multilayer on titanium coatings. Journal of The Royal Society Interface 10: 20130070. 10.1098/rsif.2013.0070 PMC3673146 23635490

[30] Fang M, Goldstein EL, Matich EK, Orr BG, Holl MM (2013) Type I collagen self-assembly: the roles of substrate and concentration. Langmuir 29: 2330–2338. 23339654 10.1021/la3048104

[31] Nassif N, Gobeaux Fdr, Seto J, Belamie E, Davidson P, et al (2010) Self-Assembled Collagen−Apatite Matrix with Bone-like Hierarchy. Chemistry of Materials 22: 3307–3309.

[32] Castellani C, Lindtner RA, Hausbrandt P, Tschegg E, Stanzl-Tschegg SE, et al (2011) Bone–implant interface strength and osseointegration: Biodegradable magnesium alloy versus standard titanium control. Acta Biomater 7: 432–440. 20804867 10.1016/j.actbio.2010.08.020

[33] Kim HW, Li LH, Lee EJ, Lee SH, Kim HE (2005) Fibrillar assembly and stability of collagen coating on titanium for improved osteoblast responses. Journal of Biomedical Materials Research Part A 75: 629–638. 16106439 10.1002/jbm.a.30463

[34] Ao H, Xie Y, Tan H, Yang S, Li K, et al.. (2013) Fabrication and in vitro evaluation of stable collagen/hyaluronic acid biomimetic multilayer on titanium coatings. Journal of The Royal Society Interface 10. 10.1098/rsif.2013.0070 PMC3673146 23635490

[35] Chen X, Ergun A, Gevgilili H, Ozkan S, Kalyon DM, et al (2013) Shell-core bi-layered scaffolds for engineering of vascularized osteon-like structures. Biomaterials 34: 8203–8212. 23896002 10.1016/j.biomaterials.2013.07.035

[36] Wu L, Luthringer BJ, Feyerabend F, Schilling AF, Willumeit R (2014) Effects of extracellular magnesium on the differentiation and function of human osteoclasts. Acta Biomaterialia 10: 2843–2854. 24531013 10.1016/j.actbio.2014.02.010

[37] Gelse K, Pöschl E, Aigner T (2003) Collagens–structure, function, and biosynthesis. Advanced drug delivery reviews 55: 1531–1546. 14623400 10.1016/j.addr.2003.08.002

[38] Kadler K, Holmes D, Trotter J, Chapman J (1996) Collagen fibril formation. Biochem J 316: 1–11. 8645190 10.1042/bj3160001 PMC1217307

[39] Ferreira AM, Gentile P, Chiono V, Ciardelli G (2012) Collagen for bone tissue regeneration. Acta Biomaterialia 8: 3191–3200. 22705634 10.1016/j.actbio.2012.06.014

[40] Cisneros DA, Hung C, Franz CM, Muller DJ (2006) Observing growth steps of collagen self-assembly by time-lapse high-resolution atomic force microscopy. Journal of structural biology 154: 232–245. 16600632 10.1016/j.jsb.2006.02.006

[41] Narayanan B, Gilmer GH, Tao J, De Yoreo JJ, Ciobanu CV (2014) Self-assembly of collagen on surfaces: the interplay of collagen-collagen and collagen-substrate interactions. Langmuir 30: 1343–1350. 24437511 10.1021/la4043364

[42] Loo RW, Goh MC (2008) Potassium ion mediated collagen microfibril assembly on mica. Langmuir 24: 13276–13278. 18973309 10.1021/la803041v

[43] Leow WW, Hwang W (2011) Epitaxially guided assembly of collagen layers on mica surfaces. Langmuir 27: 10907–10913. 21740026 10.1021/la2018055

[44] Zheng Y, Gu X, Witte F (2014) Biodegradable metals. Materials Science and Engineering: R: Reports 77: 1–34.

[45] Wang L, Guo Y, Li P, Song Y (2014) Anion-Specific Effects on the Assembly of Collagen Layers Mediated by Magnesium Ion on Mica Surface. The Journal of Physical Chemistry B. 10.1021/jp405035x 24369856

[46] He L, Cai S, Wu B, Mu C, Zhang G, et al (2012) Trivalent chromium and aluminum affect the thermostability and conformation of collagen very differently. Journal of inorganic biochemistry 117: 124–130. 23085592 10.1016/j.jinorgbio.2012.08.017

[47] Zhao N, Workman B, Zhu D (2014) Endothelialization of Novel Magnesium-Rare Earth Alloys with Fluoride and Collagen Coating. International journal of molecular sciences 15: 5263–5276. 24670478 10.3390/ijms15045263 PMC4013562

[48] Ferreira A, González G, González-Paz R, Feijoo J, Lira-Olivares J, et al (2009) Bone collagen role in piezoelectric mediated remineralization. Acta Microscopica 18: 278–286.

[49] Walter R, Kannan MB (2011) Influence of surface roughness on the corrosion behaviour of magnesium alloy. Materials & Design 32: 2350–2354.

[50] Walter R, Kannan MB, He Y, Sandham A (2013) Effect of surface roughness on the in vitro degradation behaviour of a biodegradable magnesium-based alloy. Applied Surface Science 279: 343–348.

[51] Huang H-H, Ho C-T, Lee T-H, Lee T-L, Liao K-K, et al (2004) Effect of surface roughness of ground titanium on initial cell adhesion. Biomolecular engineering 21: 93–97. 15567102 10.1016/j.bioeng.2004.05.001

[52] Matschegewski C, Staehlke S, Loeffler R, Lange R, Chai F, et al (2010) Cell architecture–cell function dependencies on titanium arrays with regular geometry. Biomaterials 31: 5729–5740. 20434213 10.1016/j.biomaterials.2010.03.073

[53] Leikina E, Mertts M, Kuznetsova N, Leikin S (2002) Type I collagen is thermally unstable at body temperature. Proceedings of the National Academy of Sciences 99: 1314–1318. 10.1073/pnas.032307099 PMC122187 11805290

[54] Reznikov N, Almany-Magal R, Shahar R, Weiner S (2013) Three-dimensional imaging of collagen fibril organization in rat circumferential lamellar bone using a dual beam electron microscope reveals ordered and disordered sub-lamellar structures. Bone 52: 676–683. 23153959 10.1016/j.bone.2012.10.034

[55] Kadler KE, Hill A, Canty-Laird EG (2008) Collagen fibrillogenesis: fibronectin, integrins, and minor collagens as organizers and nucleators. Current opinion in cell biology 20: 495–501. 18640274 10.1016/j.ceb.2008.06.008 PMC2577133

